# DNA Aptamers in the Diagnosis and Treatment of Human Diseases

**DOI:** 10.3390/molecules201219739

**Published:** 2015-11-25

**Authors:** Qinchang Zhu, Ge Liu, Masaaki Kai

**Affiliations:** 1 Faculty of Pharmaceutical Sciences, Graduate School of Biomedical Sciences, Nagasaki University, 1-14 Bunkyo-machi, Nagasaki 852-8521, Japan; zhu_qinchang@hotmail.com; 2 Department of Genomic Epidemiology, Research Center for Environment and Developmental Medical Sciences, Kyushu University, 3-1-1 Maidashi, Fukuoka 812-8582, Japan; lggege_15@hotmail.com

**Keywords:** DNA aptamers, human diseases, diagnosis, therapeutics

## Abstract

Aptamers have a promising role in the field of life science and have been extensively researched for application as analytical tools, therapeutic agents and as vehicles for targeted drug delivery. Compared with RNA aptamers, DNA aptamers have inherent advantages in stability and facility of generation and synthesis. To better understand the specific potential of DNA aptamers, an overview of the progress in the generation and application of DNA aptamers in human disease diagnosis and therapy are presented in this review. Special attention is given to researches that are relatively close to practical application. DNA aptamers are expected to have great potential in the diagnosis and treatment of human diseases.

## 1. Introduction

Aptamers can be broadly defined as short biomolecules like oligonucleotides and peptides that bind to specific targets with extremely high affinity based on their structural conformations. Since the early 1990s, systematic evolution of ligands by exponential enrichment (SELEX) and similar methods have been reported to efficiently select RNA and DNA aptamers [1,2,3,4]. Thereafter, nucleic acid aptamers have been extensively researched and applied. Nucleic acid aptamers are RNA and single-stranded (ss) DNA oligonucleotides with lengths typically ranging from 15 to 70 mers, which have the same level of target-binding affinity as monoclonal antibodies (the dissociation constant (*K*_d_) usually ranges from 0.1 to 50 nM) [5,6]. Compared with antibodies, nucleic acid aptamers have many advantages in their suitability for clinical application and industrialization, including almost no immunogenicity, efficient penetration, less batch variation, easy modification, cost-effectiveness and short production times [5,7]. In the past 25 years, much progress has been made in the use of nucleic acid aptamers, particularly RNA aptamers, as therapeutic agents, diagnostic and analytical tools, vehicles for targeted drug delivery, biosensors and even genetic control devices [5,8,9,10,11]. Until now, although only one aptamer (Macugen, Pfizer/Eyetech) has been approved for the therapeutic use in the clinic [12], ten other aptamers are being evaluated at different stages of clinical trials [5,13]. Currently, the application in diagnostics, research and development is expected to account for the largest share of the aptamer market. The global aptamer market was estimated to be valued at $107.56 million in 2015 and to reach $244.93 million by 2020 [14]. DNA aptamers are expected to account for the largest share of the global market in 2015 [14], which indicates an increase in the application of DNA aptamers.

DNA and RNA aptamers are functionally similar but have some differences in their stability and accessibility. Compared with DNA aptamers, RNA aptamers are chemically unstable because of the presence of a reactive hydroxyl group (–OH) at the 2′ position of the ribose sugar in RNA nucleotides. This –OH group easily gets deprotonated in solution, especially in alkaline solutions. The resulting anionic 2′-O^−^ may nucleophilically attack the phosphorus atom of the phosphodiester linkage, leading to the hydrolysis of RNA molecules [15]. The nuclease resistance of RNA aptamers was found to increase when the 2′-hydroxyl group was removed from the sugars of RNA [16,17]. DNA aptamers are less reactive and relatively stable because of the C–H bonds at the 2′ position of the deoxyribose sugar of DNA nucleotides. This chemical difference gives DNA aptamers an inherent advantage in stability over RNA aptamers. Our previous study also confirmed that DNA aptamers are much more stable than natural RNA aptamers in 10% fetal bovine serum (FBS) and human serum [18]. That is the reason why extra chemical modifications are usually added to RNA aptamers to improve their chemical stability [19]. However, it should be noted that the reactivity of RNA nucleotides and non-Watson-Crick base pairing makes RNA oligonucleotides prone to forming more diverse and complex three-dimensional (3D) structures [20,21], which is helpful for selecting aptamers with high affinity and specificity from RNA libraries [22]. Therefore, DNA aptamers are usually selected from the libraries containing longer randomized regions for the purpose of obtaining more complicated structures. Another potential advantage of DNA aptamers over RNA aptamers is their relatively simple selection process. The selection of RNA aptamers requires reverse transcription and *in vitro* transcription in every round of selection, as well as an initial transcription for generating the RNA library from a DNA library in most cases [1,23]. But the selection process of DNA aptamers does not require these extra steps [4,24]. Moreover, once selected, the cost of producing DNA aptamers is lower than that for RNA aptamers.

Since the first ssDNA aptamer was selected for human thrombin in 1992 [4], DNA aptamers have been researched and applied in various fields, especially in diagnosis and treatment of human diseases. DNA aptamers are usually explained to work in a similar way to RNA aptamers. Because of the potential advantages described above, DNA aptamers have gained increasing attention in recent years. However, they have seldom been reviewed systematically and independently. To better understand the unique potential of DNA aptamers, this article will review the recent progress of DNA aptamers in regard to their preparation and application in the diagnosis and treatment of human diseases. The advantages and remaining challenges to develop and use DNA-aptamer-based diagnostic tools and therapeutics will also be discussed.

## 2. Generation of DNA Aptamers

### 2.1. Conventional Method for Generating DNA Aptamers

SELEX, an interactive *in vitro* selection procedure, is the basic method used to engineer aptamers, which was first reported for screening of RNA aptamers independently by Ellington’s and Tuerk’s groups 25 years ago [1,2]. SELEX was then adapted to generate DNA aptamers [3,4]. In principle, aptamers are selected from an initial random ssDNA pool based on the binding of the oligonucleotides to the target molecules under optimal conditions. The ssDNA pool contains 10^14^–10^15^ random sequences of synthetic DNA (the typical length is 15–70 nucleotides, flanked by two constant regions with primer sites for polymerase chain reaction [PCR] amplification). The unbound sequences are separated from the bound molecules, and the target-bound sequences are amplified by PCR. The amplified products (double stranded DNA) are converted to ssDNA through various ways [25] and then used as a new aptamer pool for the next selection round. Enriched aptamer sequences are finally cloned and identified by sequencing. Usually after 10–20 rounds of selection, the specific aptamers with the strongest affinity for the target molecules are obtained.

### 2.2. Evolution of Methods for DNA Aptamers Generation

#### 2.2.1. Generation of DNA Aptamers against Unpurified Target Molecules and Live Organisms

**Figure 1:**
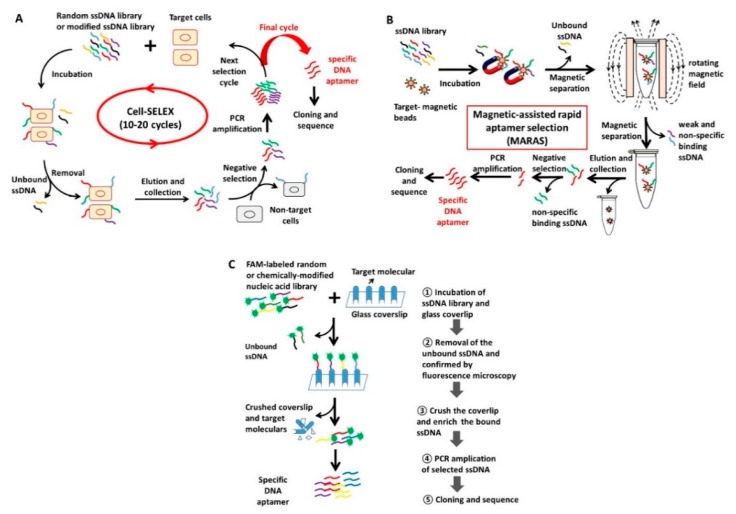
Evolution of methods for DNA aptamers generation. (**A**) Cell-SELEX: The random ssDNA pool is first incubated with target cells on ice. After washing, the bound DNAs are eluted by heating to 95 °C. The eluted DNAs are then incubated with non-target cells for negative selection. Those that do not bind to negative targets are retained and amplified by PCR. The PCR products are separated into ssDNA for further rounds. After 10–20 cycles, the selected ssDNAs are cloned and sequenced for aptamer identification; (**B**) Magnetic-assisted rapid aptamer selection (MARAS): The target-coated magnetic beads are incubated with the ssDNA pool. The beads with bound sequences are then separated from the unbound sequences with a U-shaped magnet or magnetic stand. After re-dispersion, the beads are put in an externally applied rotating or alternating magnetic field. During this process, the weak and non-specific binding sequences are released and separated. The strong-binding sequences are finally released from the beads by heating and then incubated with the beads without a target for the negative selection. The selected sequences are amplified, cloned and sequenced as usual; (**C**) One-step Selection: A FAM-labeled oligonucleotide library is incubated with a target immobilized on a glass coverslip that was coated with *N*-hydroxysuccinimide (NHS) functionalized polyethylene glycol (PEG). Unbound sequences are discarded by extensive washing followed by monitoring with fluorescence microscopy. The coverslip is later crushed and the bound sequences are eluted by heating in water. The selected aptamers are amplified, cloned and sequenced as usual.

Selection of aptamers using conventional *in vitro* SELEX requires purified and soluble target proteins. The processes used to obtain purified protein targets are time-consuming. Sometimes it is difficult to purify target proteins. Moreover, sometimes the aptamers selected using a non-native protein do not interact with the protein in a native conformation. To solve these problems, a strategy using whole live cells as targets for aptamer selection has been developed, which is known as cell-SELEX. This SELEX is able to generate DNA aptamers that recognize the cell-surface or intracellular target protein in their native conformation, which shows great potential in cell-specific therapeutics and diagnostic applications [26,27,28]. This method first selects the specific aptamers that bind to the target cells by a positive selection step, and then eliminates the non-target cell-specific aptamers by a negative selection step using non-target cells, followed by the SELEX process as summarized in Figure 1A. Recently, modified cell-SELEX methods have been developed to select aptamers targeting specific cells like disease state cells and metastatic cells. They are stimulus-response cell-SELEX [29] and metastatic-cell-based SELEX [30]. To shorten the time of conventional cell-SELEX, a two-step stimulus-response cell-SELEX method has been developed, which utilizes asymmetric PCR or streptavidin-biotin magnetic separation for the generation of single-stranded DNA to reduce the selection cycle to two steps [29]. Competitive cell-SELEX is another approach to improve selection efficiency and affinity, which utilizes a nitrocellulose membrane that contains the target cells and negative control cells to select target aptamers [31]. This method can reduce the selection time, since the negative selection step is not required.

#### 2.2.2. Novel Methods for Rapid Selection of Highly Specific DNA Aptamers

The conventional SELEX procedure is time-consuming and requires several steps to obtain a specific DNA aptamer. To effectively generate highly specific aptamers, several novel methods using different selection approaches have been developed. Combining SELEX with other method is one of the most effective ways to further improve the selection efficiency and binding affinity of DNA aptamers. For example, cell-SELEX coupled with *in silico* maturation was developed to improve aptamer specificity [31]. The method consists of cell-SELEX, a post-SELEX *in silico* process and *in vitro* screening. After the normal cell-SELEX, an extra *in silico* process was performed to further evolve improved aptamers from the SELEX-selected sequences through successive rounds of sequence shuffling and random mutation based on a genetic algorithm. This is followed by *in vitro* functional screening and selection of enhanced aptamers from the shuffling and mutated sequences. This method permits the evolution of functionally enhanced aptamer sequences recognizing targets of interest. In addition to SELEX-like methods, other methods have also been developed; magnetic-assisted rapid aptamer selection (MARAS) is a method that uses magnetic beads and an externally applied rotating magnetic field to provide the competitive mechanism for the rapid selection of aptamers with different affinity to the molecular target as summarized in Figure 1B [32,33]. This method uses biofunctionalized magnetic nanoparticles to separate target-bound DNA oligonucleotides from a library, selecting those interactions that survive a disruptive force generated by the movement of the particles in an externally applied rotating magnetic field. It abandons the multi-cycle evolutionary process used in conventional SELEX and thus can achieve rapid selection (completed in less than one hour). A one-step selection approach is another promising way to increase the rate of aptamer generation. Recently, this rapid one-step selection method was developed to select specific DNA aptamers using only one PCR step as summarized in Figure 1C [34]. In this method, a target immobilized on a glass coverslip was subjected to carboxyfluorescein (FAM)-labeled nucleic acid pool binding, extensive washing and microscopy examination, followed by PCR enrichment of the selected aptamers. A control experiment used a labeled target and a labeled DNA library was used to make sure the specificity of the selection. In the overlay image of the immobilized target labeled with Alexa Fluor 555 (red color) and target bound FAM-labeled DNA aptamer (green fluorescence), an orange color is observed for target-specific selection. Although the binding affinity of aptamers selected with the current version of the one-step method was in the low micromolar range, it was believed that it can be further improved by using larger targets, increasing the stringency of selection, and by combining it with a capillary electrophoresis separation. This method was described as a user-friendly, low-cost and easy way to select DNA aptamers. In particular, this method allows the use of a chemically modified nucleic acid library directly as it requires only one PCR step. Moreover, a DNA microarray has been utilized to achieve one-step aptamer identification, in which the sequences of interest can be produced on the arrays for selection and PCR, cloning and sequencing are not required [35,36].

#### 2.2.3. Strategies to Overcome Rapid Degradation of DNA Aptamers by Nuclease

DNA aptamers are well known to be more stable than RNA aptamers, which allows them to be readily used in the primary stage of developing diagnostic tools. However, the degradation of DNA aptamers by nuclease is still a serious problem that limits their clinical application, when they are subjected to complicated biological samples. To improve the nuclease resistance of DNA aptamers, one of the effective solutions is to use a library with chemically modified DNA sequences in the screening process. The modified DNA library can be prepared by PCR amplification using specific polymerase and catalysis reactions using modified DNA enzymes [37,38]. Modification of the aptamers can also be performed after the *in vitro* selection from natural nucleic acid library. Modifications of the sugar phosphate backbone or the pyrimidine are the strategies used to increase the stability and nuclease resistance of aptamers [38,39,40]. Capping the end of aptamers is also utilized to enhance aptamer stability against nucleases [41]. Incorporation of unnatural nucleotides is another approach to overcome aptamer instability. Locked nucleic acid (LNA) is one of the most prominent and successful nucleic acid analogues because of their pronounced stability [42,43,44]. Generation of “mirror aptamers”, which are also known as spiegelmers, is another approach to improve aptamer stability against nucleases [45].

#### 2.2.4. Strategies to Improve the Lifetime DNA Aptamer

Generation of highly specific aptamers with a long lifetime is very important for their therapeutic application. Because most DNA aptamers have a small molecular weight (ranging from 5–15 kDa), DNA aptamers are readily removed via renal filtration and metabolic processes, limiting their therapeutic application. One of the effective ways to control the lifetime of DNA aptamer *in vivo* is to conjugate the aptamers with bioavailable materials. Conjugation of DNA aptamers with polyethylene glycol (PEG) is commonly used to prolong their circulation in the bloodstream [46,47]. Coating DNA aptamers with other nanomaterials such as nanoparticles [48,49], liposomes [50] and copolymers [51] has been successfully used to improve the lifetime of DNA aptamers.

## 3. Diagnostic Application of DNA Aptamers

Once selected to bind to disease-related biomarkers or pathogens, DNA aptamers can be developed as biosensors through chemical modification with luminophores or linkage to nanoparticles in various formats [52]. For example, in an earlier study, DNA aptamers selected for *Bacillus* anthracic spores were developed to detect anthrax spores in an aptamer–magnetic bead-electrochemiluminescence (AM-ECL) sandwich mode [53]. The Dynal M-280 magnetic beads were covered with the aptamer and used as the capturer to capture the spores, while another biotinylated aptamer was used as the reporter and the signal was finally transduced through the streptavidin-Ru(bpy)_3_^2+^ ECL. Since the first DNA aptamer was selected, dozens of DNA aptamers have been selected for the use of disease-related detection. Although there are no aptamer-based diagnostic tools that are in clinical use at the moment, many preclinical studies indicate that DNA aptamers have great potential to be used in this way. To profile the features of recent studies that use DNA aptamers as a diagnostic tool for human diseases, we listed some DNA aptamers selected for this purpose in Table 1, with a special attention to parameters like the sensitivity and specificity, which have a great impact on their potential for clinical use.

### 3.1. Diseases and Biomarkers

In theory, aptamers can be selected for developing diagnostic tools for various diseases, as long as definite targets, such as disease-specific biomarkers or pathogens, are available. To date, DNA aptamers have been explored mainly for the diagnosis of infectious diseases, cancer and cardiovascular diseases. Because of the convenience of cell-SELEX, which does not require preparation and purification of target molecules, many reported DNA aptamers are selected with cancer cells, aiming to diagnose and image cancer tissue. These cancer cells include pancreatic [54], colon [30], liver [55,56], cholangio [57], gastric [58,59], prostate [27], breast [60] and glioblastoma [61] cancer cells. Usually, the selection consists of positive selection with cancer cells and negative selection with normal cells from the same organs, as described in Section 2.2.3. It is worth noting that, because the diagnosis and monitoring of metastasis of cancer cells is important for the treatment of cancer, several DNA aptamers have been selected with metastatic cancer cells and negatively selected with non-metastatic cancer cells [27,30,60].

**Table 1:** Examples of selected DNA aptamers for the diagnosis of human diseases.

Aptamer		Target	Length in Random Region (mer)	*K*_d_ (nM)	Diagnostic Mode	Sensitivity	Specificity	Refs.
**Cancers**								
Wy-5a		Prostate cancer cells (PC-3, metastatic)	45	73.59	Direct binding (FITC labeled)	ns	100% ( *n* = 7 cancer cell lines)	[27]
XL-33		Metastatic colon cancer cells (SW620)	45	0.7	Direct binding (FAM labeled)	81.7% (*n* = 71 metastatic colon cancer tissues)	66.7% (*n* = 18 nonmetastatic colon cancer tissues)	[30]
XQ-2d		Pancreatic ductal adenocarcinomas (PDAC) cells (PL45)	42	55.02	Direct binding (Cy5-labeled)	82.5% (*n* = 40 PDAC tissues)	75% (*n* = 8 normal pancreatic tissues)	[54]
C-2		Liver cancer cells (HepG2)	50	19	Direct binding (FITC labeled)	ns.	ns.	[55]
JHIT2		Liver cancer cells (HepG2)	25	64	Direct binding (FAM labeled)	ns.	ns.	[56]
yl19		Cholangiocarcinoma cells (QBC-939)	40	42.4	Direct binding (FAM labeled)	ns.	100% (*n* = 6 cancer cell lines)	[57]
AGC03		Gastric cancer cells (HGC-27)	40	16.5	Direct binding (FAM labeled)	ns.	Recognized different gastric cancer cells but not liver cancer cells	[58]
Cy-apt20		Gastric carcinoma cells (AGS)	52	ns.	Direct binding (FAM/FITC labeled)	70%	ns.	[59]
LXL-1		Metastatic breast cancer cells (MDA-MB-231)	45	44.0	Direct binding (Cy5 labeled)	76% (*n* = 34)	100% ( *n* = 8 cancer cell lines)	[60]
GBM128		Glioblastoma cells (U118-MG)	45	20	Direct binding (Cy5 labeled)	ns.	80% (*n* = 10 cancer tissues)	[61]
32		Glioblastoma multiforme cells (U87Δ) epidermal growth factor receptor variant III (EGFRvIII)	30	0.62	Direct binding (FITC labeled)	ns.	ns.	[62]
SYL3-C		Solid cancer epithelial cell adhesion molecule (EpCAM)	40	22.8	Direct binding (FITC labeled)	60%	100% (*n* = 3)	[63]
Vea5 (SL2-B)		Cancer cells biomarker: vascular endothelial growth factor (VEGF165)	30	130 (0.5)	Direct binding (PE-texas red-labeled)	ns.	ns.	[64,65]
GMT3		Glioblastoma multiforme cells (A172)	42	75.3	Direct binding (biotin-labeled, streptavidin–PE reported)	ns.	87.5% (*n* = 8 cancer cell lines)	[66]
**Infectious Diseases**								
Sequence (2)	HA protein (H5N1)		74	4.65	Dot blot (streptavidin-alkaline phosphatase)	1.28 HAU (hemagglutinating unit)	100% (*n* = 5 avian influenza virus subtypes)	[67]
LmWC-25R and LmHSP-7b/11R	Leishmania promastigote and hydrophilic surface protein (HSP)		36	ns.	Aptamer-magnetic bead sandwich assay (HRP)	100 ng (parasite protein)	ns.	[68]
4C6	Truncated murine prion protein (H-MoPrP_90-231_)		45	20	Target-induced dissociation	13.0 nmol/L	ns.	[69,70]
2008s	Plasmodium falciparum lactate dehydrogenase (PfLDH)		35	42–59	Direct binding (AuNP-labeled)	57 ng/mL	No human LDH recognition	[71]
**Cardiovascular Diseases**								
Myo040-7-27	Myoglobin		40	4.93	Target-induced dissociation (RuHex)	10 pm	ns.	[72]
Hcy8	l-homocysteine		60	600	Target-induced dissociation (AuNP)	0.5 μM	100% (*n* = 3 amino acids)	[73]

ns.: non-specified.

For example, Li *et al.* selected DNA aptamers for metastatic colon cancer cells using SW620 cells derived from a metastatic site lymph node in the positive selection and SW480 cells from a primary colon adenocarcinoma of the same patient in the negative selection [30]. The resulting aptamer (XL-33) was found to possess specific affinity to the metastatic colon cancer cells (*K*_d_ = 0.7 nM). Its truncated form (XL-33-1) was used to image the cancer tissue after labeling with fluorescein amidite (FAM), displaying an 81.7% detection rate against colon cancer tissue with metastasis in regional lymph nodes and 66.7% specificity against nonmetastatic colon cancer tissue.

The biomarker molecules that specifically express or overexpress on the cancer cells were also used for the selection of diagnostic DNA aptamers. In addition to the previously known prostate-specific membrane antigen (PSMA) [74] and mucin 1 (MUC1) [75], other biomarkers like epidermal growth factor receptor variant III (EGFRvIII) [62], epithelial cell adhesion molecule (EpCAM) [63] and vascular endothelial growth factor (VEGF165) [64] have also been used to select DNA aptamers for cancer detection. By directly using cancer biomarkers to select cancer-cell-recognized aptamers, you obtain highly specific and affinitive aptamers, but the potential risk is that the aptamers might not recognize the natural biomarkers on the surface of cancer cells because of the difference in the 3D structure of the purified biomarkers and the natural biomarkers.

For the diagnosis of infectious agents, lots of DNA aptamers have been selected predominantly with cell-SELEX and magnetic beads-based methods by targeting various viruses and bacteria or their antigen protein [6], including targets such as norovirus, influenza virus, severe acute respiratory syndrome coronavirus (SARS-CoV), hepatitis C virus (HCV), hepatitis B virus (HBV), human immunodeficiency virus (HIV), human papillomavirus (HPV), salmonella typhimurium, and pathogenic *E. coli*. Because of the low cost and short production time of DNA aptamers, the DNA aptamer based diagnostic tools hold particular advantages for the diagnosis of infections that need point-of-care testing, such as infections caused by highly pathogenic pandemic influenza virus, HIV, SARS-CoV and malaria. Avian influenza virus H5N1 is a highly pathogenic subtype of the influenza virus, which can also infect humans and has a high mortality rate. Wang *et al.* selected a DNA aptamer targeting H5N1 using both the virus antigen and the whole virus particle using the SELEX method [67]. For the first four selection cycles, the purified virus antigen hemagglutinin (HA) was used as the target protein, while for the remaining eight cycles the entire H5N1 virus particles were used as the targets. The selected aptamer showed high affinity *(K*_d_ = 4.6 nM) and specificity to the H5N1 virus (Table 1). Using such a mixed selection mode might be a good strategy to obtain ideal aptamers, which can overcome the drawbacks of selection using only free biomarkers or cells.

In the case of cardiovascular diseases, markers in the blood, such as myoglobin, C-reactive protein, l-homocysteine and thrombin have been used to select DNA aptamer for the diagnosis of related diseases. Myoglobin increases after acute myocardial infarction, which is an important early marker in urgent diagnosis of cardiovascular diseases. Wang *et al.* selected DNA aptamers against myoglobin using a fluidic chip method [72]. The DNA aptamer with the lowest *K*_d_ value (4.93 nM) was subjected to the development of different biosensors for the detection of myoglobin, including the Myoglobin-induced structural switching supersandwich biosensor [72] and the antibody-Myoglobin–aptamer sandwich biosensor [76]. Human C-reactive protein (CRP) is a homopentameric oligoprotein, which has been validated as a powerful predictor and risk factor of inflammation and cardiovascular disease [77]. Yang *et al.* selected a DNA aptamer (*K*_d_ = 3.9 nM) targeting CRP and used it to develop a sensor based on surface plasmon resonance technology [78]. l-Homocysteine is an amino acid intermediate, whose elevated level in the blood is associated with coronary heart disease [79]. McKeague *et al.* selected DNA aptamers targeting l-homocysteine and developed an aptamer-AuNP sensor, in which the DNA aptamers first coil around the surface of the gold particles and release the particles after binding to the homocysteine, which leads to salt-induced aggregation and colorization of the particles [73]. Thrombin is a serine protease that plays a critical role in the formation of obstructive blood clots, or thrombosis, which is involved in various diseases including chronic cardiovascular diseases [80]. The first reported DNA aptamer is the thrombin aptamer. Since then, thrombin aptamers have been used as model aptamers to explore the mode of aptamer-based biosensors. It is the most frequently used DNA aptamer for the demonstration of the proof-of-principle of various detection methods [81].

### 3.2. Diagnostic Modes

**Figure 2:**
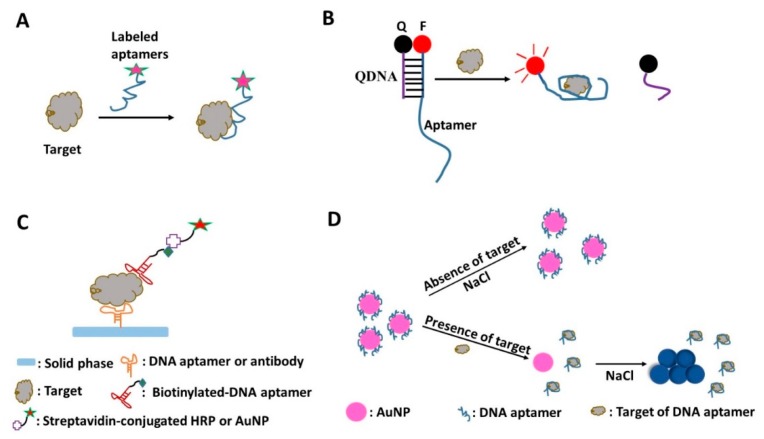
Scheme showing examples of the modes used in DNA aptamer-based detection. (**A**) Direct binding-based mode: The signal-molecule-labeled aptamers directly bind to the immobilized or free target. The signal molecules on the target are detected directly; (**B**) Target-induced structural switching mode: A fluorophore-labeled DNA aptamer forms a partial duplex with a small oligonucleotide modified with a quenching moiety (QDNA) in the absence of the target, bringing the fluorophore (F) and the quencher (Q) into close proximity for maximum fluorescence quenching. When the target is introduced, the aptamer prefers to form the aptamer-target complex. The binding of the target to the aptamer will change the structure of the aptamers and release the partially complementary quencher, which will trigger the increase of the signal; (**C**) Sandwich-like mode: The aptamer or antibody is immobilized on the solid phase as the capturer. The captured target is reported by the biotinylated aptamer, which displays the signal through further binding to the streptavidin-conjugated HRP or AuNP; (**D**) Target-induced dissociation mode: DNA aptamers coil around the surface of the gold nanoparticles (AuNPs) and stop the salt-induced aggregation of AuNPs. When the target appears, the DNA aptamers bind to the target and release from the AuNPs. The AuNPs without the cover of aptamers aggregate in the salt solution and change color from red to purple.

The general modalities and assay formats for using aptamers in sensors have been well summarized in many comprehensive reviews [82,83,84,85,86]. They can be roughly classified into four types based on the way the signal is generated: (1) direct binding-based mode; (2) target-induced structural switching mode; (3) sandwich-like mode; and (4) target-induced assembly or dissociation mode. These protocols are illustrated in Figure 2. In the direct binding-based mode (Figure 2A), aptamers labeled with signal molecules directly bind to the target. The signal molecules on the aptamer-bound target can be detected directly. The aptamers for cancer cells imaging usually use this mode (Table 1). It is a simple and direct mode, which is especially suitable for the situations like *in vivo* imaging that do not allow complex detection. But it does not contain signal amplification, which makes it difficult to increase the detection sensitivity. In target-induced structural switching mode, the binding of the target will cause a specific conformational change to the aptamer, which will “switch on or switch off” the signal generation [87,88]. For example, in a bio-chromophoric target-induced structural switching approach, a fluorophore-labeled DNA aptamer forms a partial duplex with a small oligonucleotide modified with a quenching moiety (denoted QDNA) in the absence of the target, bringing the fluorophore (F) and the quencher (Q) into close proximity for maximum fluorescence quenching. When the target is introduced, the aptamer prefers to form the aptamer-target complex. The binding of the target to the aptamer will change the structure of the aptamers and release the partially complementary quencher, which will trigger the increase of the signal (Figure 2B). This mode is suited for the development of aptamer-based reporters for real-time sensing applications. But the limitation of this mode is the decreased aptamer binding affinity because of competition from the QDNA [87]. In the sandwich-like mode, the target is “the meat” inserted within “two pieces of bread” consisting of an aptamer/aptamer or antibody/aptamer, which is very similar to the enzyme-linked immunosorbent assay (ELISA) (Figure 2C). Aptamers in this mode function as reporter and/or capturer. This method permits double recognition of the target and signal amplification, which can greatly improve the specificity and sensitivity of aptamer-based detection. Moreover, because of the small size of the aptamer, the aptamer sandwich mode can conquer the potential limitation of antibody ELISA in detecting small molecules [89]. But this mode requires extensive washing during the detection. In the target-induced assembly or dissociation mode, binding of the target to the aptamer will trigger the assembly of split aptamers or dissociation of bound aptamers, along with the change of detectable signals [82,90] (Figure 2D). In addition, it should be noted that various detection methods have been applied by DNA aptamer based biosensors, including electrochemical, chemiluminescence, fluorescence, colorimetric, quantum dots-based and mass-sensitive detections [84].

For imaging of cancer cells, most of the DNA aptamer based methods use fluorophore-labeled aptamers to recognize the cancer cells directly (Table 1). For the small targets like microorganisms and the marker protein, methods based on different modes have been explored extensively. The sandwich-like mode is frequently used (Figure 2C) [68,91]. In the example of *Leishmania* detection [68], the aptamer (LmWC-25R) targeting the promastigote of *Leishmania* was immobilized on the surface of the M280 magnetic beads. After the target *Leishmania* promastigote were captured by LmWC-25R, another 5′-biotinylated reporter aptamer (LmHSP-7b/11R) that targets the hydrophilic surface protein (HSP) of *Leishmania* promastigote was added. Then the beads with aptamer-captured *Leishmania* promastigote and reporters were collected on the magnetic rack. Streptavidin–horseradish peroxidase (Sav-HRP) and the substrate Ample Red were added to transduce and display the signal. Another frequently used mode is the target-induced dissociation mode, in which the system was in a non-signal status until the single-stranded aptamer dissociated from the initial state as a result of binding to the target [69,72,73]. The detection of l-homocysteine mentioned above is a good example of the target-induced dissociation mode [73] (Figure 2D). DNA aptamers coil around the surface of the gold nanoparticles (AuNPs) and prevent the salt-induced aggregation of AuNPs. When the target molecules appear, the DNA aptamers fold into a rigid structure and bind to the targets, and therefore release from the AuNPs. The AuNPs without the coverage of aptamers aggregate in the salt solution and change color from red to purple.

### 3.3. Test with Clinical Samples and in Vivo Study

One of the biggest challenges for aptamer-based diagnostic tools and their clinical use is the possible invalidation in a real biological environment as a result of degradation from nucleases or interference from other matrix factors. Using chemical modified DNA aptamers is an effective way to prolong the lifetime of DNA aptamer in the biological samples. For ultimate success, sufficient studies with clinical samples or *in vivo* studies are indispensable. Currently, although most of the studies about DNA aptamer based diagnostic tools for human diseases are still in the primary stage of laboratory studies, some of them have gone further by testing them with clinical samples and animal studies *in vivo* [30,54,59,61]. The DNA aptamer XQ-2d (Table 1) that targets the pancreatic ductal adenocarcinomas (PDAC) cells has been tested with clinical PDAC tissue sections and *in vivo* imaging of PDAC tumor-bearing mice [54]. 33 out of 40 PDAC tissues and two out of eight normal pancreatic tissues were detected by the Cy5-labeled XQ-2d. In the *in vivo* imaging test, Cy5-labeled XQ-2d was injected into the BALB/c-nude mice grafted with PDAC through the tail vein. It was found to illuminate the tumor site up to 3 h post-injection, while the Cy5-labeled library control did not give any signal. For this study, both phosphorothioate and 2′-*O*-methyl modifications were tried for the DNA aptamer. The four bases at the 5′ and 3′ termini of XQ-2d were replaced by ether phosphorothioate oligonucleotides or 2′-*O*-methyl oligonucleotides. However, the phosphorothioate modification was found to weaken the binding ability of the aptamer, whereas the 2′-*O*-methyl modification did not significantly affect the binding ability. Moreover, the 2′-*O*-methyl modified XQ-2d could last for 36 h in 10% FBS medium, but the unmodified XQ-2d was completely degraded after 24-h incubation.

## 4. DNA Aptamers as Therapeutic Agents

An important application of aptamers is their use as small-molecule therapeutic agents. DNA aptamers exhibit significant advantages in therapeutic application and have been developed as attractive therapeutic agents in competition with antibodies. Generally, DNA aptamers used for therapeutic applications function in two ways. First, DNA aptamers can inhibit protein-protein interactions by specifically binding to the target protein and thereby functioning as antagonists. Second, DNA aptamers can function as agonists, which promotes the function of the target protein upon binding to the target protein. Although DNA aptamers act similarly to antibodies, the non-immunogenicity of DNA aptamers make it more notable in therapeutics. Compared with antibodies, DNA aptamers are easier to uptake because of their small size. Importantly, aptamers can specifically recognize a wide range of targets including small molecules, proteins and cells. Moreover, given that DNA aptamers can be designed and selected *in vitro*, they have lower production cost than antibodies. The properties and advantages of DNA aptamers mentioned above facilitate the promising application of DNA aptamers in the field of therapeutics.

### 4.1. DNA Aptamers as Therapeutic Agents in Clinical Trials and Preclinical Laboratory Studies

Although there are currently no DNA aptamer therapeutics in clinical use, four DNA aptamers are being evaluated in clinical trials for their effect on hematological disease, macular degeneration disease and cancer (Table 2).

AS1411 (Antisoma) is a guanine-rich aptamer with a guanine quadruplex structure, targeting nucleolin. Nucleolin is a eukaryotic nucleolar phosphoprotein that is involved in the synthesis and maturation of ribosomes and has been reported as a target for anti-cancer therapies. The guanine quadruplex structure can help to enhance the nuclease degradation resistance and cell uptake of AS1411. AS1411 possesses anti-cancer activity against breast cancer cells [92], metastatic renal cell carcinoma [93] and acute myeloid leukemia [94]. The phase 2 clinical trial for using AS1411 to treat renal cell carcinoma was completed in 2009 (NCT00740441), while another phase 2 clinical trial for treating acute myeloid leukemia was completed in 2011 (NCT01034410 and NCT01034410).

ARC1779 (Achemix), a PEGylated DNA aptamer, recognizes platelet ligand receptor von Willebrand factor that mediates platelet recruitment. ARC1779 blocks the binding between von Willebrand factor and the platelet, thereby inducing an antithrombotic effect. The efficacy of ARC1779 in platelet inhibition has been demonstrated and a phase 2 clinical trial for treating von Willebrand factor related platelet function disorders (NCT00632242) has been completed. Recent studies show that ARC1779 can effectively prevent thromboembolism [95]. A phase 2 clinical study shows that ARC1779 exhibits favorable pharmacokinetic, pharmacodynamic and safety properties in patients with congenital thrombotic thrombocytopenic purpura [96].

NU172 (ARCA biopharma) is an unmodified DNA aptamer targeting thrombin, which can prolong blood clotting. The phase 2 clinical trial using NU172 as anticoagulation agent in patients undergoing off-pump coronary artery bypass graft (CABG) surgery (SNAP-CABG-OFF) has been completed (NCT00808964).

E10030 (Ophthotech), a PEGylated DNA aptamer, functions as an antagonist of platelet-derived growth factor. E10030 in combination with anti-VEGF agent can effectively prevent angiogenesis [97]. Currently, a phase 3 clinical study of E10030 in combination with ranibizumab (Lucentis^®^) for wet age-related macular degeneration treatment is ongoing (NCT01944839).

**Table 2:** DNA aptamers in clinical investigation.

Aptamer	Developer	Type	Target	Clinical Trial	Treated Disease
AS1411	Antisoma	G-rich DNA aptamer	Nucleolin	Phase 2 (completed)	Renal cell carcinoma [93] and acute myeloid leukemia [94,98]
ARC1779	Achemix	PEGylated DNA aptamer	von Willebrand factor	Phase 2 (completed)	Thromboembolism [95], congenital thrombotic thrombocytopenic purpura [96], and von Willebrand disease [99]
NU172	ARCA biopharma	Non-chemically modified DNA aptamer	Thrombin	Phase 2 (completed)	Off-pump coronary artery bypass graft surgery (ClinicalTrials.gov Identifier: NCT00808964)
E10030	Ophthotech	PEGylated DNA aptamer	Platelet-derived growth factor	Phase 3 (undergoing)	Age-related macular degeneration (ClinicalTrials.gov Identifier: NCT01944839)

Besides the DNA aptamers in clinical trials, many promising DNA aptamers are in preclinical studies for treating various diseases, such as virus infections, tumors and central nervous system diseases. A DNA aptamer recognizing the receptor-binding region of influenza A hemagglutinin was found to inhibit viral infection in an animal model against different influenza strains, as manifested by a 90%–99% reduction of the virus burden in the lungs of treated mice [100]. DNA aptamer NAS-24 was found to bind to vimentin and then cause apoptosis of mouse ascites adenocarcinoma cells *in vitro* and *in vivo* [101]. Recently, DNA aptamers targeting human epidermal growth factor receptor 2 (ErbB-2/HER2) was demonstrated to retard the tumorigenic growth of gastric cancer in mice with more effective activity than anti-ErbB-2/HER2 monoclonal antibody [102]. Moreover, remyelination was induced by a DNA aptamer in a mouse model of multiple sclerosis (inflammatory disease of the central nervous system), which highlights the potential therapeutic application of DNA aptamers in the treatment of multiple sclerosis [103].

### 4.2. DNA Aptamers for Targeted Delivery of Drugs 

Cell-specific drug delivery can help to increase the efficiency of a drug and reduce side effects. In addition to functioning as therapeutic agents, DNA aptamers have also been explored as delivery vehicles in targeted delivery of drugs or small oligonucleotides such as small interference RNA (siRNA) and microRNA (miRNA). The ability of aptamers to specifically recognize the target and to be readily modified makes it a potential targeted delivery tool. DNA aptamers are used in two ways for targeted delivery of drugs: (1) directly linked to the drug molecules; (2) in combination with nanoparticles to form the delivery platform.

Conjugating drug molecules directly to specific DNA aptamers is a potential way to deliver the drugs specifically, and thus reduce the risk of off-target drugs. By linking DNA aptamers to drugs or packing the drug into an aptamer-folded structure, DNA aptamers–drug conjugates can efficiently deliver drugs to target cells with increased specificity. Many DNA aptamers have been selected to efficiently deliver chemotherapy drugs *in vitro* or *in vivo*, such as doxorubicin (DOX) [104], fluorouracil [105] and epirubicin [106]. Dimeric or dendrimer DNA aptamers in conjugation with drugs have been developed to further enhance the efficiency of target delivery [107,108]. With regard to small oligonucleotides delivery, DNA aptamers were directly linked to the small oligonucleotides to form a DNA aptamer-oligonucleotide chimera, which could help to prevent non-specific internalization as well as decrease the cellular toxicity towards non-target cells. We have reported that a DNA aptamer-siRNA chimera could specifically enter into CD4 (+) T cells and efficiently decrease the expression of exogenous the HIV protease gene [18]. An anti-mucin 1 DNA aptamer covalently linked to miRNA-29b was found to deliver miRNA-29b into ovarian cancer cells specifically and induce significant apoptosis of the cancer cells [109].

DNA aptamers in combination with nanoparticles as a delivery vehicle is another promising targeted delivery approach. A number of nanomaterials have been explored to conjugate with DNA aptamers to form the delivery platform [110,111]. DNA aptamer conjugated liposome likely has the highest potential as a delivery system, and liposome-based drug delivery systems have been evaluated in clinical trials. For example, AS1411 aptamer conjugated liposome was found be able to enhance the delivery specificity and uptake of DOX in tumor cells, as well as to increase the accumulation of DOX in the tumor tissues with reduced cardiotoxicity *in vivo* [112]. Micelles, aggregation of lipid molecules, are also used in DNA aptamer-nanoparticles delivery systems. Recently, AS1411 aptamer conjugated PEG-poly(lactic-co-glycolic acid) (PLGA) nanoparticles have been developed to facilitate antiglioma delivery of paclitaxel *in vivo* [49]. This aptamer-PEG-PLGA delivery system can prolong circulation time and enhance target accumulation of paclitaxel, which facilitates tumor inhibition. Gold nanoparticles are another attractive material used in drug delivery system, because of their high stability, low or no toxicity and facile conjugation. A DNA aptamer (sgc8c) conjugated gold nanoparticle system has been found to increase the uptake of DOX into cancer cells [113]. Other nanomaterials such as silica, carbon nanotubes and quantum dots have also been used in DNA aptamer–nanoparticles delivery systems to enhance the specificity and prolong the circulation of drug molecules [114].

## 5. Future Perspectives

By summarizing the progress of the generation of DNA aptamers and their application in human disease diagnosis and therapy, we have shown that DNA aptamers have great potential to be used as an alternative to antibodies. Since the first monoclonal antibody was produced in 1970s, antibodies have been successfully and extensively used in the diagnosis and therapy of human diseases. Aptamers are expected to achieve a similar success to antibodies. Because of their stability, low cost and facile manipulation, DNA aptamers will continue to be extensively studied and applied. Although modified RNA aptamers have enhanced stability, the high cost of chemically modified RNA might limit their study and application. However, RNA aptamers hold an advantage in providing more complex and diverse 3D structures, which is helpful for selecting aptamers with high affinity for complex targets needed for disease therapy. Therefore, DNA aptamers might have more promising application in diagnosis and *in vivo* imaging, while modified RNA aptamers might have more promising application in therapy. In the era of personalized medicine, DNA aptamer-based therapeutics and diagnostics are believed to have great potential for extensive application because of their flexibility to specifically bind to any molecule targets. Before they can be widely applied, there are still many problems that remain to be addressed. Problems like nuclease degradation, quick renal excretion and potential cross-reactivity of aptamers should be analyzed in future studies.

## References

[1] Tuerk C., Gold L. (1990). Systematic evolution of ligands by exponential enrichment: RNA ligands to bacteriophage T4 DNA polymerase. Science.

[2] Ellington A.D., Szostak J.W. (1990). *In vitro* selection of RNA molecules that bind specific ligands. Nature.

[3] Ellington A.D., Szostak J.W. (1992). Selection *in vitro* of single-stranded DNA molecules that fold into specific ligand-binding structures. Nature.

[4] Bock L.C., Griffin L.C., Latham J.A., Vermaas E.H., Toole J.J. (1992). Selection of single-stranded DNA molecules that bind and inhibit human thrombin. Nature.

[5] Keefe A.D., Pai S., Ellington A. (2010). Aptamers as therapeutics. Nat. Rev. Drug Discov..

[6] Hong K.L., Sooter L.J. (2015). Single-Stranded DNA Aptamers against Pathogens and Toxins: Identification and Biosensing Applications. Biomed. Res. Int..

[7] Sun H., Zu Y. (2015). A Highlight of Recent Advances in Aptamer Technology and Its Application. Molecules.

[8] Song K.M., Lee S., Ban C. (2012). Aptamers and their biological applications. Sensors.

[9] Huang R.R., Xi Z.J., He N.Y. (2015). Applications of aptamers for chemistry analysis, medicine and food security. Sci. China-Chem..

[10] Tsae P.K., DeRosa M.C. (2015). Outlook for Aptamers after Twenty Five Years. Curr. Top. Med. Chem..

[11] Berens C., Groher F., Suess B. (2015). RNA aptamers as genetic control devices: The potential of riboswitches as synthetic elements for regulating gene expression. Biotechnol. J..

[12] Vavvas D., D’Amico D.J. (2006). Pegaptanib (Macugen): Treating neovascular age-related macular degeneration and current role in clinical practice. Ophthalmol. Clin. N. Am..

[13] Lao Y.H., Phua K.K., Leong K.W. (2015). Aptamer nanomedicine for cancer therapeutics: Barriers and potential for translation. ACS Nano.

[14] Markets R.A. Aptamers Market-Global Forecast to 2020. http://www.researchandmarkets.com/research/6b92mp/aptamers_market2015.

[15] Barciszewski J., Clark B.F.C. (2012). RNA Biochemistry and Biotechnology.

[16] Adler A., Forster N., Homann M., Goringer H.U. (2008). Post-SELEX chemical optimization of a trypanosome-specific RNA aptamer. Comb. Chem. High Throughput Screening.

[17] Wilson C., Keefe A.D. (2006). Building oligonucleotide therapeutics using non-natural chemistries. Curr. Opin. Chem. Biol..

[18] Zhu Q., Shibata T., Kabashima T., Kai M. (2012). Inhibition of HIV-1 protease expression in T cells owing to DNA aptamer-mediated specific delivery of siRNA. Eur. J. Med. Chem..

[19] Wang R.E., Wu H., Niu Y., Cai J. (2011). Improving the Stability of Aptamers by Chemical Modification. Curr. Med. Chem..

[20] Fohrer J., Hennig M., Carlomagno T. (2006). Influence of the 2′-hydroxyl group conformation on the stability of A-form helices in RNA. J. Mol. Biol..

[21] Schneider B., Morávek Z., Berman H.M. (2004). RNA conformational classes. Nucleic Acids Res..

[22] Sun H., Zhu X., Lu P.Y., Rosato R.R., Tan W., Zu Y. (2014). Oligonucleotide Aptamers: New Tools for Targeted Cancer Therapy. Mol. Ther. Nucleic Acids.

[23] Wilson D.S., Szostak J.W. (1999). *In vitro* selection of functional nucleic acids. Annu. Rev. Biochem..

[24] Hernandez L.I., Machado I., Schafer T., Hernandez F.J. (2015). Aptamers Overview: Selection, Features and Applications. Curr. Top. Med. Chem..

[25] Marimuthu C., Tang T.H., Tominaga J., Tan S.C., Gopinath S.C. (2012). Single-stranded DNA (ssDNA) production in DNA aptamer generation. Analyst.

[26] Ara M.N., Hyodo M., Ohga N., Hida K., Harashima H. (2012). Development of a novel DNA aptamer ligand targeting to primary cultured tumor endothelial cells by a cell-based SELEX method. PLoS ONE.

[27] Wang Y., Luo Y., Bing T., Chen Z., Lu M., Zhang N., Shangguan D., Gao X. (2014). DNA aptamer evolved by cell-SELEX for recognition of prostate cancer. PLoS ONE.

[28] Meyer S., Maufort J.P., Nie J., Stewart R., McIntosh B.E., Conti L.R., Ahmad K.M., Soh H.T., Thomson J.A. (2013). Development of an efficient targeted cell-SELEX procedure for DNA aptamer reagents. PLoS ONE.

[29] Ji K., Lim W.S., Li S.F., Bhakoo K. (2013). A two-step stimulus-response cell-SELEX method to generate a DNA aptamer to recognize inflamed human aortic endothelial cells as a potential *in vivo* molecular probe for atherosclerosis plaque detection. Anal. Bioanal. Chem..

[30] Li X., An Y., Jin J., Zhu Z., Hao L., Liu L., Shi Y., Fan D., Ji T., Yang C.J. (2015). Evolution of DNA aptamers through *in vitro* metastatic-cell-based systematic evolution of ligands by exponential enrichment for metastatic cancer recognition and imaging. Anal. Chem..

[31] Savory N., Lednor D., Tsukakoshi K., Abe K., Yoshida W., Ferri S., Jones B.V., Ikebukuro K. (2013). In silico maturation of binding-specificity of DNA aptamers against Proteus mirabilis. Biotechnol. Bioeng..

[32] Lai J.-C., Hong C.-Y. (2014). A novel protocol for generating high-affinity ssDNA aptamers by using alternating magnetic fields. J. Mater. Chem. B.

[33] Lai J.C., Hong C.Y. (2014). Magnetic-assisted rapid aptamer selection (MARAS) for generating high-affinity DNA aptamer using rotating magnetic fields. ACS Comb. Sci..

[34] Lauridsen L.H., Shamaileh H.A., Edwards S.L., Taran E., Veedu R.N. (2012). Rapid one-step selection method for generating nucleic acid aptamers: Development of a DNA aptamer against alpha-bungarotoxin. PLoS ONE.

[35] Knight C.G., Platt M., Rowe W., Wedge D.C., Khan F., Day P.J., McShea A., Knowles J., Kell D.B. (2009). Array-based evolution of DNA aptamers allows modelling of an explicit sequence-fitness landscape. Nucleic Acids Res..

[36] Martin J.A., Mirau P.A., Chushak Y., Chavez J.L., Naik R.R., Hagen J.A., Kelley-Loughnane N. (2015). Single-Round Patterned DNA Library Microarray Aptamer Lead Identification. J. Anal. Methods Chem..

[37] Latham J.A., Johnson R., Toole J.J. (1994). The application of a modified nucleotide in aptamer selection: Novel thrombin aptamers containing 5-(1-pentynyl)-2′-deoxyuridine. Nucleic Acids Res..

[38] Kuwahara M., Sugimoto N. (2010). Molecular evolution of functional nucleic acids with chemical modifications. Molecules.

[39] King D.J., Ventura D.A., Brasier A.R., Gorenstein D.G. (1998). Novel combinatorial selection of phosphorothioate oligonucleotide aptamers. Biochemistry.

[40] Kusser W. (2000). Chemically modified nucleic acid aptamers for *in vitro* selections: Evolving evolution. J. Biotechnol..

[41] Kasahara Y., Kitadume S., Morihiro K., Kuwahara M., Ozaki H., Sawai H., Imanishi T., Obika S. (2010). Effect of 3′-end capping of aptamer with various 2′,4′-bridged nucleotides: Enzymatic post-modification toward a practical use of polyclonal aptamers. Bioorg. Med. Chem. Lett..

[42] Schmidt K.S., Borkowski S., Kurreck J., Stephens A.W., Bald R., Hecht M., Friebe M., Dinkelborg L., Erdmann V.A. (2004). Application of locked nucleic acids to improve aptamer *in vivo* stability and targeting function. Nucleic Acids Res..

[43] Orum H., Wengel J. (2001). Locked nucleic acids: A promising molecular family for gene-function analysis and antisense drug development. Curr. Opin. Mol. Ther..

[44] Elle I.C., Karlsen K.K., Terp M.G., Larsen N., Nielsen R., Derbyshire N., Mandrup S., Ditzel H.J., Wengel J. (2015). Selection of LNA-containing DNA aptamers against recombinant human CD73. Mol. Biosyst..

[45] Urata H., Ogura E., Shinohara K., Ueda Y., Akagi M. (1992). Synthesis and properties of mirror-image DNA. Nucleic Acids Res..

[46] Takafuji Y., Jo J.I., Tabata Y. (2010). Simple PEG Modification of DNA Aptamer Based on Copper Ion Coordination for Tumor Targeting. J. Biomater. Sci. Polym. Ed..

[47] Mallikaratchy P.R., Ruggiero A., Gardner J.R., Kuryavyi V., Maguire W.F., Heaney M.L., McDevitt M.R., Patel D.J., Scheinberg D.A. (2011). A multivalent DNA aptamer specific for the B-cell receptor on human lymphoma and leukemia. Nucleic Acids Res..

[48] Tan L., Neoh K.G., Kang E.T., Choe W.S., Su X. (2012). Affinity analysis of DNA aptamer-peptide interactions using gold nanoparticles. Anal. Biochem..

[49] Guo J., Gao X., Su L., Xia H., Gu G., Pang Z., Jiang X., Yao L., Chen J., Chen H. (2011). Aptamer-functionalized PEG-PLGA nanoparticles for enhanced anti-glioma drug delivery. Biomaterials.

[50] Xing H., Tang L., Yang X., Hwang K., Wang W., Yin Q., Wong N.Y., Dobrucki L.W., Yasui N., Katzenellenbogen J.A. (2013). Selective Delivery of an Anticancer Drug with Aptamer-Functionalized Liposomes to Breast Cancer Cells *in vitro* and *in vivo*. J. Mater. Chem. B.

[51] Wu Y., Sefah K., Liu H., Wang R., Tan W. (2010). DNA aptamer-micelle as an efficient detection/delivery vehicle toward cancer cells. Proc. Natl. Acad. Sci. USA.

[52] Hong P., Li W., Li J. (2012). Applications of Aptasensors in Clinical Diagnostics. Sensors.

[53] Bruno J.G., Kiel J.L. (1999). *In vitro* selection of DNA aptamers to anthrax spores with electrochemiluminescence detection. Biosens. Bioelectron..

[54] Wu X., Zhao Z., Bai H., Fu T., Yang C., Hu X., Liu Q., Champanhac C., Teng I.T., Ye M. (2015). DNA Aptamer Selected against Pancreatic Ductal Adenocarcinoma for *in vivo* Imaging and Clinical Tissue Recognition. Theranostics.

[55] Ninomiya K., Kaneda K., Kawashima S., Miyachi Y., Ogino C., Shimizu N. (2013). Cell-SELEX based selection and characterization of DNA aptamer recognizing human hepatocarcinoma. Bioorg. Med. Chem. Lett..

[56] Xu J.H., Teng I.T., Zhang L.Q., Delgado S., Champanhac C., Cansiz S., Wu C.C., Shan H., Tan W.H. (2015). Molecular Recognition of Human Liver Cancer Cells Using DNA Aptamers Generated via Cell-SELEX. PLoS ONE.

[57] Wan J., Ye L., Yang X.H., Guo Q.P., Wang K.M., Huang Z.X., Tan Y.Y., Yuan B.Y., Xie Q. (2015). Cell-SELEX based selection and optimization of DNA aptamers for specific recognition of human cholangiocarcinoma QBC-939 cells. Analyst.

[58] Zhang X.J., Zhang J., Ma Y.Y., Pei X.Y., Liu Q.M., Lu B., Jin L., Wang J.C., Liu J. (2014). A cell-based single-stranded DNA aptamer specifically targets gastric cancer. Int. J. Biochem. Cell Biol..

[59] Cao H.Y., Yuan A.H., Chen W., Shi X.S., Miao Y. (2014). A DNA aptamer with high affinity and specificity for molecular recognition and targeting therapy of gastric cancer. BMC Cancer.

[60] Li X.L., Zhang W.Y., Liu L., Zhu Z., Ouyang G.L., An Y., Zhao C.Y., Yang C.J. (2014). *In Vitro* Selection of DNA Aptamers for Metastatic Breast Cancer Cell Recognition and Tissue Imaging. Anal. Chem..

[61] Kang D.Z., Wang J.J., Zhang W.Y., Song Y.L., Li X.L., Zou Y., Zhu M.T., Zhu Z., Chen F.Y., Yang C.J. (2012). Selection of DNA Aptamers against Glioblastoma Cells with High Affinity and Specificity. PLoS ONE.

[62] Tan Y., Shi Y.S., Wu X.D., Liang H.Y., Gao Y.B., Li S.J., Zhang X.M., Wang F., Gao T.M. (2013). DNA aptamers that target human glioblastoma multiforme cells overexpressing epidermal growth factor receptor variant III *in vitro*. Acta Pharmacol. Sin..

[63] Song Y.L., Zhu Z., An Y., Zhang W.T., Zhang H.M., Liu D., Yu C.D., Duan W., Yang C.J. (2013). Selection of DNA Aptamers against Epithelial Cell Adhesion Molecule for Cancer Cell Imaging and Circulating Tumor Cell Capture. Anal. Chem..

[64] Kaur H., Yung L.Y. (2012). Probing high affinity sequences of DNA aptamer against VEGF165. PLoS One.

[65] Hasegawa H., Sode K., Ikebukuro K. (2008). Selection of DNA aptamers against VEGF(165) using a protein competitor and the aptamer blotting method. Biotechnol. Lett..

[66] Bayrac A.T., Sefah K., Parekh P., Bayrac C., Gulbakan B., Oktem H.A., Tan W.H. (2011). *In Vitro* Selection of DNA Aptamers to Glioblastoma Multiforme. ACS Chem. Neurosci..

[67] Wang R., Zhao J., Jiang T., Kwon Y.M., Lu H., Jiao P., Liao M., Li Y. (2013). Selection and characterization of DNA aptamers for use in detection of avian influenza virus H5N1. J. Virol. Methods.

[68] Bruno J.G., Richarte A.M., Phillips T., Savage A.A., Sivils J.C., Greis A., Mayo M.W. (2014). Development of a fluorescent enzyme-linked DNA aptamer-magnetic bead sandwich assay and portable fluorometer for sensitive and rapid leishmania detection in sandflies. J. Fluoresc..

[69] Xiao S.J., Hu P.P., Xiao G.F., Wang Y., Liu Y., Huang C.Z. (2012). Label-free detection of prion protein with its DNA aptamer through the formation of T-Hg2+-T configuration. J. Phys. Chem. B.

[70] Bibby D.F., Gill A.C., Kirby L., Farquhar C.F., Bruce M.E., Garson J.A. (2008). Application of a novel *in vitro* selection technique to isolate and characterise high affinity DNA aptamers binding mammalian prion proteins. J. Virol. Methods.

[71] Cheung Y.W., Kwok J., Law A.W., Watt R.M., Kotaka M., Tanner J.A. (2013). Structural basis for discriminatory recognition of Plasmodium lactate dehydrogenase by a DNA aptamer. Proc. Natl. Acad. Sci. USA.

[72] Wang Q., Liu W., Xing Y., Yang X., Wang K., Jiang R., Wang P., Zhao Q. (2014). Screening of DNA Aptamers against Myoglobin Using a Positive and Negative Selection Units Integrated Microfluidic Chip and Its Biosensing Application. Anal. Chem..

[73] McKeague M., Foster A., Miguel Y., Giamberardino A., Verdin C., Chan J.Y., DeRosa M.C. (2013). Development of a DNA aptamer for direct and selective homocysteine detection in human serum. RSC Adv..

[74] Jolly P., Formisano N., Estrela P. (2015). DNA aptamer-based detection of prostate cancer. Chem. Pap..

[75] Ferreira C.S.M., Papamichael K., Guilbault G., Schwarzacher T., Gariepy J., Missailidis S. (2008). DNA aptamers against the MUC1 tumour marker: Design of aptamer-antibody sandwich ELISA for the early diagnosis of epithelial tumours. Anal. Bioanal. Chem..

[76] Wang Q., Liu F., Yang X., Wang K., Wang H., Deng X. (2015). Sensitive point-of-care monitoring of cardiac biomarker myoglobin using aptamer and ubiquitous personal glucose meter. Biosen. Bioelectron..

[77] Shrivastava A.K., Singh H.V., Raizada A., Singh S.K. (2015). C-reactive protein, inflammation and coronary heart disease. Egypt. Heart J..

[78] Yang X., Wang Y., Wang K., Wang Q., Wang P., Lin M., Chen N., Tan Y. (2014). DNA aptamer-based surface plasmon resonance sensing of human C-reactive protein. RSC Adv..

[79] Humphrey L.L., Fu R., Rogers K., Freeman M., Helfand M. (2008). Homocysteine level and coronary heart disease incidence: A systematic review and meta-analysis. Mayo Clinic Proceedings.

[80] Tracy R.P. (2003). Thrombin, inflammation, and cardiovascular disease: An epidemiologic perspective. Chest.

[81] Deng B., Lin Y., Wang C., Li F., Wang Z., Zhang H., Li X.F., Le X.C. (2014). Aptamer binding assays for proteins: The thrombin example—A review. Anal. Chim. Acta.

[82] Han K., Liang Z., Zhou N. (2010). Design strategies for aptamer-based biosensors. Sensors.

[83] Zhou J., Battig M.R., Wang Y. (2010). Aptamer-based molecular recognition for biosensor development. Anal. Bioanal. Chem..

[84] Iliuk A.B., Hu L., Tao W.A. (2011). Aptamer in bioanalytical applications. Anal. Chem..

[85] Xing H., Hwang K., Li J., Torabi S.F., Lu Y. (2014). DNA Aptamer Technology for Personalized Medicine. Curr. Opin. Chem. Eng..

[86] Zhou W., Huang P.J., Ding J., Liu J. (2014). Aptamer-based biosensors for biomedical diagnostics. Analyst.

[87] Nutiu R., Li Y. (2003). Structure-switching signaling aptamers. J. Am. Chem. Soc..

[88] Nutiu R., Li Y. (2004). Structure-switching signaling aptamers: Transducing molecular recognition into fluorescence signaling. Chemistry.

[89] Sharma A.K., Kent A.D., Heemstra J.M. (2012). Enzyme-Linked Small-Molecule Detection Using Split Aptamer Ligation. Anal. Chem..

[90] Liu X., Shi L., Hua X., Huang Y., Su S., Fan Q., Wang L., Huang W. (2014). Target-induced conjunction of split aptamer fragments and assembly with a water-soluble conjugated polymer for improved protein detection. ACS Appl. Mater. Interfaces.

[91] Yang H., Ji J., Liu Y., Kong J., Liu B. (2009). An aptamer-based biosensor for sensitive thrombin detection. Electrochem. Commun..

[92] Soundararajan S., Chen W., Spicer E.K., Courtenay-Luck N., Fernandes D.J. (2008). The nucleolin targeting aptamer AS1411 destabilizes Bcl-2 messenger RNA in human breast cancer cells. Cancer Res..

[93] Rosenberg J.E., Bambury R.M., Van Allen E.M., Drabkin H.A., Lara P.N., Harzstark A., Wagle N., Figlin R.A., Smith G.W., Garraway L.A. (2014). A phase II trial of AS1411 (a novel nucleolin-targeted DNA aptamer) in metastatic renal cell carcinoma. Investig. New Drugs.

[94] Stuart R.K., Stockerl-Goldstein K., Cooper M., Devetten M., Herzig R., Medeiros B., Schiller G., Wei A., Acton G., Rizzieri D. (2009). Randomized phase II trial of the nucleolin targeting aptamer AS1411 combined with high-dose cytarabine in relapsed/refractory acute myeloid leukemia (AML). J. Clin. Oncol..

[95] Markus H.S., McCollum C., Imray C., Goulder M.A., Gilbert J., King A. (2011). The von Willebrand inhibitor ARC1779 reduces cerebral embolization after carotid endarterectomy: A randomized trial. Stroke.

[96] Jilma-Stohlawetz P., Gilbert J.C., Gorczyca M.E., Knobl P., Jilma B. (2011). A dose ranging phase I/II trial of the von Willebrand factor inhibiting aptamer ARC1779 in patients with congenital thrombotic thrombocytopenic purpura. Thromb. Haemost..

[97] Ni Z., Hui P. (2009). Emerging pharmacologic therapies for wet age-related macular degeneration. Ophthalmologica.

[98] Mongelard F., Bouvet P. (2010). AS-1411, a guanosine-rich oligonucleotide aptamer targeting nucleolin for the potential treatment of cancer, including acute myeloid leukemia. Curr. Opin. Mol. Ther..

[99] Cosmi B. (2009). ARC-1779, a PEGylated aptamer antagonist of von Willebrand factor for potential use as an anticoagulant or antithrombotic agent. Curr. Opin. Mol. Ther..

[100] Jeon S.H., Kayhan B., Ben-Yedidia T., Arnon R. (2004). A DNA aptamer prevents influenza infection by blocking the receptor binding region of the viral hemagglutinin. J. Biol. Chem..

[101] Zamay T.N., Kolovskaya O.S., Glazyrin Y.E., Zamay G.S., Kuznetsova S.A., Spivak E.A., Wehbe M., Savitskaya A.G., Zubkova O.A., Kadkina A. (2014). DNA-aptamer targeting vimentin for tumor therapy *in vivo*. Nucleic Acid Ther..

[102] Mahlknecht G., Maron R., Mancini M., Schechter B., Sela M., Yarden Y. (2013). Aptamer to ErbB-2/HER2 enhances degradation of the target and inhibits tumorigenic growth. Proc. Natl. Acad. Sci. USA.

[103] Nastasijevic B., Wright B.R., Smestad J., Warrington A.E., Rodriguez M., Maher L.J. (2012). Remyelination induced by a DNA aptamer in a mouse model of multiple sclerosis. PLoS ONE.

[104] Huang Y.F., Shangguan D., Liu H., Phillips J.A., Zhang X., Chen Y., Tan W. (2009). Molecular assembly of an aptamer-drug conjugate for targeted drug delivery to tumor cells. Chembiochem.

[105] Wang R., Zhu G., Mei L., Xie Y., Ma H., Ye M., Qing F.L., Tan W. (2014). Automated modular synthesis of aptamer-drug conjugates for targeted drug delivery. J. Am. Chem. Soc..

[106] Jalalian S.H., Taghdisi S.M., Hamedani N.S., Kalat S.A., Lavaee P., Zandkarimi M., Ghows N., Jaafari M.R., Naghibi S., Danesh N.M. (2013). Epirubicin loaded super paramagnetic iron oxide nanoparticle-aptamer bioconjugate for combined colon cancer therapy and imaging *in vivo*. Eur. J. Pharm. Sci..

[107] Boyacioglu O., Stuart C.H., Kulik G., Gmeiner W.H. (2013). Dimeric DNA Aptamer Complexes for High-capacity-targeted Drug Delivery Using pH-sensitive Covalent Linkages. Mol. Ther. Nucleic Acids.

[108] Zhang H., Ma Y., Xie Y., An Y., Huang Y., Zhu Z., Yang C.J. (2015). A controllable aptamer-based self-assembled DNA dendrimer for high affinity targeting, bioimaging and drug delivery. Sci. Rep..

[109] Dai F., Zhang Y., Zhu X., Shan N., Chen Y. (2012). Anticancer role of MUC1 aptamer-miR-29b chimera in epithelial ovarian carcinoma cells through regulation of PTEN methylation. Target Oncol..

[110] Yang L., Zhang X., Ye M., Jiang J., Yang R., Fu T., Chen Y., Wang K., Liu C., Tan W. (2011). Aptamer-conjugated nanomaterials and their applications. Adv. Drug Deliv. Rev..

[111] Li X., Zhao Q., Qiu L. (2013). Smart ligand: Aptamer-mediated targeted delivery of chemotherapeutic drugs and siRNA for cancer therapy. J. Control. Release.

[112] Liao Z.X., Chuang E.Y., Lin C.C., Ho Y.C., Lin K.J., Cheng P.Y., Chen K.J., Wei H.J., Sung H.W. (2015). An AS1411 aptamer-conjugated liposomal system containing a bubble-generating agent for tumor-specific chemotherapy that overcomes multidrug resistance. J. Control. Release.

[113] Latorre A., Posch C., Garcimartin Y., Celli A., Sanlorenzo M., Vujic I., Ma J., Zekhtser M., Rappersberger K., Ortiz-Urda S. (2014). DNA and aptamer stabilized gold nanoparticles for targeted delivery of anticancer therapeutics. Nanoscale.

[114] Zhou J., Rossi J.J. (2014). Cell-type-specific, Aptamer-functionalized Agents for Targeted Disease Therapy. Mol. Ther. Nucleic Acids.

